# The eHealth Enhanced Chronic Care Model: A Theory Derivation Approach

**DOI:** 10.2196/jmir.4067

**Published:** 2015-04-01

**Authors:** Perry M Gee, Deborah A Greenwood, Debora A Paterniti, Deborah Ward, Lisa M Soederberg Miller

**Affiliations:** ^1^School of NursingDivision of Health SciencesIdaho State UniversityPocatello, IDUnited States; ^2^Diabetes Program Coordinator, Sutter Medical FoundationSacramento, CAUnited States; ^3^Center for Healthcare Policy and ResearchUC Davis Health SystemUniversity of California, DavisSacramento, CAUnited States; ^4^Betty Irene Moore School of NursingUC Davis Health SystemUniversity of California, DavisSacramento, CAUnited States; ^5^Human EcologyHuman Development UnitUniversity of California, DavisDavis, CAUnited States

**Keywords:** chronic disease, mobile health, eHealth, telemedicine, personal health records, social networks, education of patients

## Abstract

**Background:**

Chronic illnesses are significant to individuals and costly to society. When systematically implemented, the well-established and tested Chronic Care Model (CCM) is shown to improve health outcomes for people with chronic conditions. Since the development of the original CCM, tremendous information management, communication, and technology advancements have been established. An opportunity exists to improve the time-honored CCM with clinically efficacious eHealth tools.

**Objective:**

The first goal of this paper was to review research on eHealth tools that support self-management of chronic disease using the CCM. The second goal was to present a revised model, the eHealth Enhanced Chronic Care Model (eCCM), to show how eHealth tools can be used to increase efficiency of how patients manage their own chronic illnesses.

**Methods:**

Using Theory Derivation processes, we identified a “parent theory”, the Chronic Care Model, and conducted a thorough review of the literature using CINAHL, Medline, OVID, EMBASE PsychINFO, Science Direct, as well as government reports, industry reports, legislation using search terms “CCM or Chronic Care Model” AND “eHealth” or the specific identified components of eHealth. Additionally, “Chronic Illness Self-management support” AND “Technology” AND several identified eHealth tools were also used as search terms. We then used a review of the literature and specific components of the CCM to create the eCCM.

**Results:**

We identified 260 papers at the intersection of technology, chronic disease self-management support, the CCM, and eHealth and organized a high-quality subset (n=95) using the components of CCM, self-management support, delivery system design, clinical decision support, and clinical information systems. In general, results showed that eHealth tools make important contributions to chronic care and the CCM but that the model requires modification in several key areas. Specifically, (1) eHealth education is critical for self-care, (2) eHealth support needs to be placed within the context of community and enhanced with the benefits of the eCommunity or virtual communities, and (3) a complete feedback loop is needed to assure productive technology-based interactions between the patient and provider.

**Conclusions:**

The revised model, eCCM, offers insight into the role of eHealth tools in self-management support for people with chronic conditions. Additional research and testing of the eCCM are the logical next steps.

## Introduction

### Background

**Figure 1:**
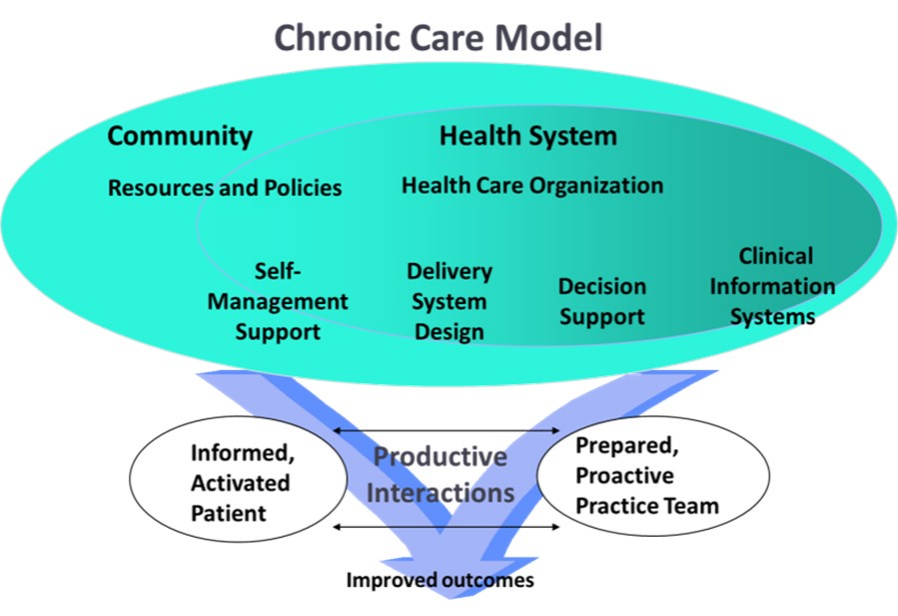
The Chronic Care Model. Developed by the MacColl Institute, ©ACP-JSIM Journals and Books, reprinted with permission from ACP-ASIM Journals and Books.

Chronic illness is a burden on individuals and society; nearly half of Americans have at least one chronic illness accounting for more than three-fourths of America’s health care spending [1,2]. The Chronic Care Model (CCM) is a well-established and validated framework that illustrates a comprehensive approach to caring for the chronically ill that supports increased functional and clinical outcomes. The model includes six key interdependent components: (1) community resources, (2) health system support, (3) self-management support, (4) delivery system design, (5) decision support, and (6) clinical information systems (Figure 1).

The CCM places chronic care in the context of the community where the person will receive health care services and with the health systems involved in that care. The CCM highlights the importance of “Self-Management Support”—giving patients the knowledge, confidence, and skills for self-management of their condition. “Delivery System Design” is also important to promote a patient-centered interdisciplinary team approach to care. “Decision Support” is needed to assure providers and patients have access to the most current and relevant evidenced-based guidelines for care, Finally, the model emphasizes the role of “Clinical Information Systems” to provide access to data, information, and knowledge needed to improve health. Effective and productive patient and provider interactions are the heart of the CCM and the key to improving outcomes [3].

Over the last decade, the CCM has been implemented and evaluated in a variety of settings in both domestic and international studies. The CCM has proven to be a useful framework for patient empowerment, self-management support, and improving clinical and behavioral outcomes [4-9]. The purpose of this review is to update the CCM with emerging eHealth technologies. This goal is consistent with the self-management support for chronic disease using technology tools suggested in both the Affordable Care Act (ACA) and the Health Information Technology for Economic and Clinical Health Act (HITECH Act) [10-12].

### eHealth for Chronic Illness

Leaders and policy makers on a global scale are strongly encouraging the use of eHealth technologies. Australia, Europe, South Korea, and the United States all have strong eHealth initiatives that are developing policy for using information technology to improve health and health care systems. The Washington think tank “eHealth Initiative” (promoting policy focused on research and education in eHealth), the Institute of Medicine (IOM), and the Agency for Healthcare Research and Quality (AHRQ) also recommend the use of eHealth as a tool to support self-management in chronic illness [13-15]. Large systematic reviews conducted by the AHRQ determined that eHealth tools can improve patient engagement and health outcomes, however, more research is needed [14,15]. Jimison et al [14] also identified that eHealth technology interventions must contain a closed or complete feedback loop (CFL) to have an impact on chronic illness outcomes. A complete feedback loop contains five stages: (1) transmission of data and information regarding the health status of the consumer, (2) interpretation of data and information using previously established knowledge and/or wisdom and use of evidence-based standards, (3) address the specific need of the individual consumer, (4) timely feedback to the consumer addressing their requirements, and (5) regular repetition of the feedback loop [14].

Despite the strong push for eHealth, there is no standard definition, which hinders research and implementation. Comprehensive systematic reviews have identified as many as 51 different definitions for eHealth in the literature [13,16-18]. Overall, the literature describes the definition of eHealth to be very broad and encompassing, ranging from the very business-oriented to more clinically focused. The authors of this paper have developed a definition of eHealth for chronic illness self-management: To promote positive health outcomes by using a new frame of mind that incorporates information and communication technologies in the presence of a complete feedback loop and enables the use of data and information, to generate health management knowledge and wisdom.

Previous eHealth definitions by Eysenbach and Eng, and the Informatics language from Staggers and Thompson [19-21] influenced the definition. In the literature, the components of eHealth typically consist of use of the Internet, telemedicine, and communication [16]. In the IOM report *Health Literacy, eHealth, and Communication: Putting the Consumer First*, the round table members noted that the eHealth Initiative was guided by Wagner’s vision of the CCM and used as the “blueprint” for eHealth to support chronic illness [13]. For enhanced use with the CCM, the authors suggest specific components highlighted in the information technology and communication literature including use of the Internet for health information, social networking, telehealth, mHealth (including wearable devices), electronic health records (EHRs), and electronic personal health records/patient portals (PHRs).

### Use of the Internet for Self-Management Support

The Internet serves as a conduit for self-management support, connecting providers and consumers to secure portals, health applications, social networks, and large databases. Roughly 80% of adults have sought health information on the Internet, including 62% of adults with a chronic illness; of those, 75% of the chronically ill surveyed stated their most recent Internet encounter affected decisions about the self-management of their condition [22-24]. The Internet is also the vehicle used by many adults for access to social networking sites.

### Social Networking or eHealth Communities

Social networking or virtual communities are newer components of eHealth. A recent study reported in JAMA regarding the diabetes online community (DOC), “TuDiabetes”, found that the use of the social network augmented hypoglycemia surveillance among the members of that virtual community [25]. To date, most research on the impact of social networks has been descriptive but there are a few studies that have shown improved health outcomes; no studies to date have shown adverse effects on consumers/patients [26]. One randomized controlled trial (RCT) followed overweight and sedentary adults and found that the use of an online community helped maintain adherence to the program and that the participants had lower attrition from the study [27]. In recent descriptive studies, virtual community members with diabetes and heart disease found that the environment was useful for asking questions, reporting personal experiences, and even supported eHealth literacy [28,29]. Social networking may be an effective tool to encourage consumer empowerment and promote patient-centered care [30,31].

### Telehealth

One well-researched component of eHealth is in the area of telehealth, sometimes called telemedicine, which has been used extensively as an intervention across many aspects of health care. Telehealth (telecommunication, videoconferencing, remote monitoring, etc) can range from performing a detailed physical examination either synchronously or asynchronously, to using videoconferencing (audio/video technology) for the delivery of a class or training to individuals or groups in a remote setting [32,33].

The telehealth field is challenged with a clear definition. A recent study by Doarn et al [34] found there are seven United States government definitions for telemedicine. A standard definition of telehealth to facilitate the use and research eHealth tools is essential. To add strength to this movement, a federal telemedicine group was commissioned, FedTel, and legislation has recently been introduced to Congress to establish federal telehealth standards [34,35].

Telehealth has been especially effective in the management of diabetes. A recent systematic review evaluating 15 RCTs described that hemoglobin A1c (A1C), a laboratory examination that measures average blood glucose over the past 2-3 months, improved when telehealth interventions incorporated more elements of structured self-monitoring of blood glucose [33]. Telehealth also lends itself to use by all members of the health care team. In a recent RCT, Tang et al [36] found that nurse-led, multi-disciplinary telehealth interventions were effective in improving A1C outcomes. In another nurse-led telehealth intervention designed for high-risk dialysis patients, the participants reported being more empowered and better able to provide needed self-management of illness [37]. In a recent RCT, Young et al found that a telehealth nurse coaching model for people with diabetes produced higher self-efficacy scores in the control group than for those who received the usual care [38]. Health care leaders, clinicians, and policymakers view telehealth as a powerful resource for improving health outcomes, health care quality, and to promote patient engagement [15,39,40].

### Mobile Health

Mobile health (mHealth) components of eHealth span a broad spectrum of technologies. mHealth includes technology that is wireless, mobile [41], or wearable (eg, sensors, medication pumps, or wristbands that monitor physical activity). mHealth also includes thousands of health apps designed for mobile devices. The market for mHealth apps is anticipated to grow 25% per year with no foreseeable end in sight [42]. mHealth is a “disruptive innovation” providing entrée to Internet-based health resources to groups who previously had barriers to these tools; 60% of Americans gain access to the Internet using a laptop, tablet computer, or mobile phone [43]. Older adults, Hispanics, and African-Americans are adopting mobile technologies at a faster rate than the general population [43]. Progress in the area of mobile phone text messaging has created a surge in using the tool for health self-management. Several recent studies and systematic reviews have reported modest health outcome improvement using text messaging as a targeted intervention [44-46].

### Electronic Health Records/Personal Health Records (EHR/PHR)

The EHR is an electronic longitudinal record of care and patient information that may be shared across multiple health care settings [47]. The tethered PHR, or patient portal, is a component of the EHR that communicates with the provider’s EHR or is integrated within the provider’s EHR and provides access to health records for patients/consumers and/or caregivers. The Markle Foundation [48] defined the PHR as “an electronic application through which individuals can access, manage and share their health information, and that of others for whom they are authorized in a private, secure and confidential environment” (p. 14)(Markle Foundation, 2008)(Markle Foundation, 2008).

Health care leaders and policy makers highly recommend PHR use as a management tool for chronic illness [49-52]. However, the PHR literature including systematic reviews related to use and health outcomes were inconclusive and the few RTCs conducted on PHR interventions failed to make the case for efficacy [53]. While the scientific evidence does not demonstrate that using PHRs can improve health outcomes, the literature does support the impact on secondary outcomes related to self-management support [36]. In an observational cohort study of 8705 subjects, Sarkar et al [54] found that patients with diabetes who use the PHR to refill their prescriptions had better medication adherence. In spite of some demonstration for the efficacy of PHRs in optimizing care, the PHR literature, including systematic reviews related to use and health outcomes, has been inconclusive [53,55].

With the national and international push to implement eHealth technologies into the current care environment, there is an opportunity to augment the established CCM with integration of eHealth technology components. The remainder of this paper will describe how adding eHealth components to the CCM may provide more self-management tools for the person with chronic illness.

## Methods

### Theory Derivation

The Theory Derivation process was used to bring together the related eHealth concepts and to grasp the relatively new phenomenon of using eHealth tools for the self-management of chronic illness [56]. Theory derivation is a structured set of procedures where one chooses a parent theory or model that is used to guide the development of a new model or theory supported by a comprehensive understanding of the current literature [56]. In this paper, the CCM was carefully examined and supporting components were extrapolated for the development of a new model. Additionally, a methodical review of a wide range of literature was conducted. A draft framework was then developed and expanded by a continued review of new literature and evaluation of the established and new components of the revised model.

A thorough review of the published literature since 2000 was conducted using CINAHL, Medline, OVID, EMBASE, PsychINFO, Science Direct, and selected “grey” literature including government reports, industry reports, legislation, etc. The review involved using the search terms “CCM or Chronic Care Model” AND “eHealth” and then we searched the specific identified components of eHealth and Chronic disease self-management support (Virtual communities”, “Virtual health communities”, “e-Communities”, “on-line communities”, social networking”, “Telemedicine”, Telehealth”, “Internet use for health”, “mHealth”, “Electronic health records”, “Personal health records”, “Patient portals”, “User training”, “Technology”, “Chronic Illness”, “Chronic disease”, and “Self-management support”). Selection criteria included review papers, randomized controlled trials, cohort studies, cross-sectional studies, and qualitative studies. The researchers independently identified papers based on framework, design, sample, measures, and fit with self-management support and chronic illness. The CCM was carefully studied in the literature and then key components of the current CCM were used to provide a framework for new model construction.

## Results

### Summary

We identified 260 papers, but excluded 63.5% (165/260) due to concerns about study design, sample size, and/or methods. Overall, with the exception of telehealth interventions, there was a noted heterogeneity of methods and approaches used. We organized the literature into the components of the CCM, highlighting the role that eHealth tools and concepts can play in each component, modifying and adding components where needed to capture the emerging eHealth literature.

### Adding the eCommunity and an Informatics Framework

**Figure 2:**
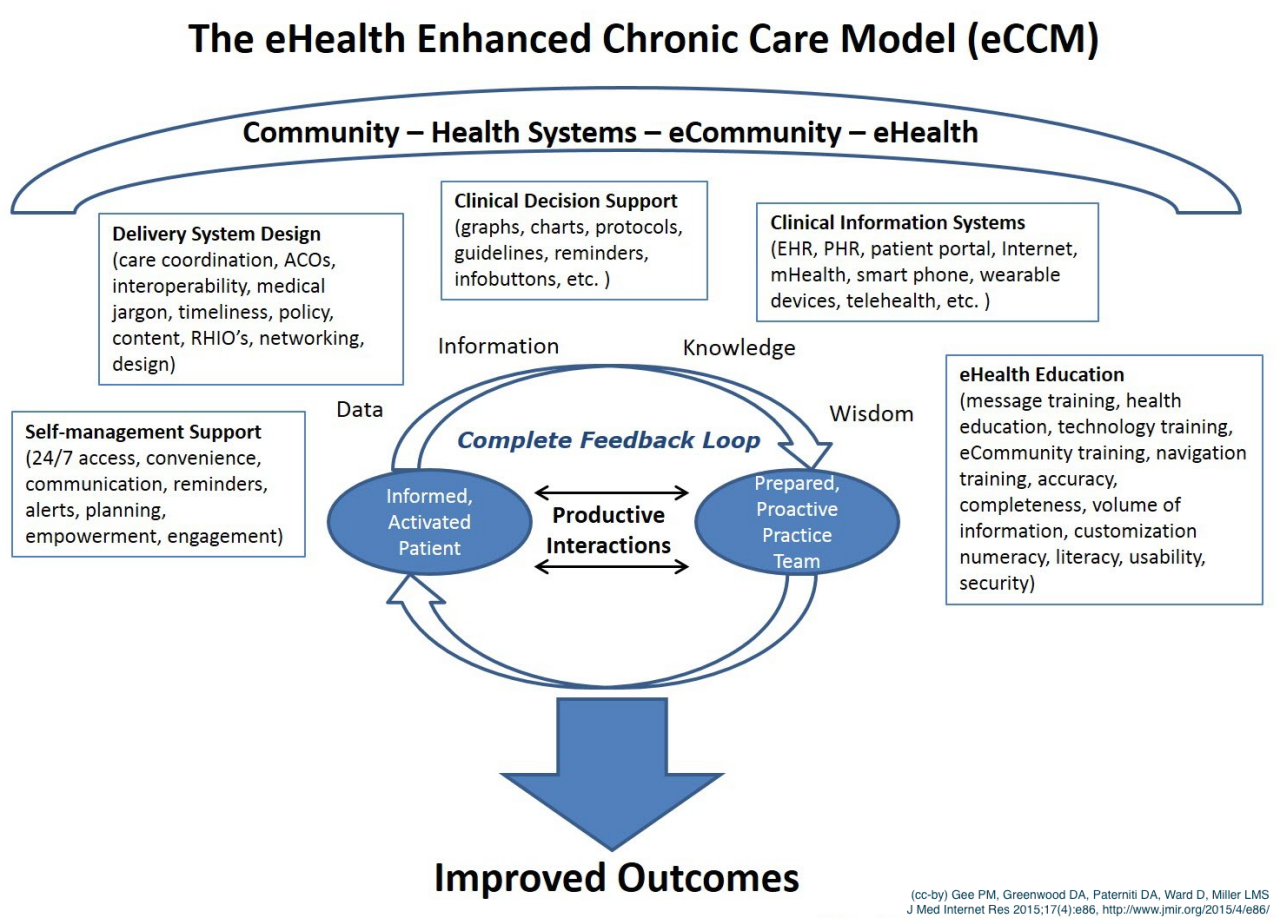
The eHealth Enhanced Chronic Care Model. Created by Gee, P M; Greenwood, D A; Paterniti, D A; Ward, D; and Miller, L M S (JMIR, 2015). Adapted from The Chronic Care Model (see Figure 1).

The CCM has two major components: Community and Health Systems (Figure 1)*.* The role of the community in the CCM is to provide support for patient engagement or activation and for self-management [57]. Based on the literature, the notion of community should be expanded to include online community and health-related social networks, or eCommunity (Figure 2). A total of 72% of American adults who use the Internet are already using social networks [58]. In a recent PEW poll, 26% of respondents stated they went online to observe the health postings about someone else’s medical condition and 16% search the Internet to find another person with the same health ailment [59,60]. Hu, Bell, Kravitz and Orrange [61] found that in a survey of 505 participants in an online support group the adult members accessed the group to prepare for upcoming medical appointments.Virtual communities including “TuDiabetes” or “PatientsLikeMe” are already supporting thousands of chronically ill adults; both groups have vigorous research activities [25,62]. Health care technology leaders and policy makers are touting a new PHR 2.0 with expected growth of 221% over the next 3 years [63]. The concept of PHR 2.0 will contain the typical components of the current PHR/patient portal systems but add social networking, gaming, and e-visits [63].

Chronically ill adults who are activated, educated, engaged, and empowered, or e-patients, are already using eHealth tools [64,65]. E-patients, together with their providers, community, and social networks, have the ability to generate a collective knowledge and wisdom about their health care self-management needs greater than any of them working alone [65,66]. Figure 2, the eHealth enhanced CCM, contains the terms data, information, knowledge, and wisdom (DIKW). These words illustrate that the DIKW framework is underpinning the process of data and information used to create new knowledge and ultimately the collective wisdom to improve health outcomes [65,66].

### Health Systems Enhancements

In the literature, it was noted that the health system must be designed to support organizations and providers and enable them to be prepared and proactive and to foster productive interactions with consumers; this is essential to improve health outcomes [67]. Kaiser Permanente and the Veterans Health Administration have made strategic efforts to implement eHealth technologies (PHRs, mHealth, telehealth, and Internet use) to improve access to care, reduce costs, and empower patients [68-71]. Between 2003 and 2010, Kaiser Permanente designed a system where patients could better manage their own health using a PHR [69,72]. Research at Kaiser showed a 25.3% reduction in the number of face-to-face office visits in primary care due to PHR use [73]. These findings suggest that health systems who consciously implement and encourage the use of eHealth technologies may achieve a higher level of patient engagement, satisfaction, and self-management support.

### Delivery System Design Enhancements

“Meaningful Use” requires redesign of health care delivery systems to meet the emerging eHealth technologies to be implemented nation-wide. Meaningful Use Requirements state that providers are eligible for up to a US $44,000 payment from the centers of Medicare and Medicaid Services to implement provider electronic health record systems [10,74]. Stage II Meaningful Use further requires that providers and health care organizations implement a patient/provider communication portal system by 2014.

Access to and control over personal health data is a theme described by some health care consumers. This is an instance where the literature identified policy change and interoperability—the exchanging of data and information electronically between health care systems—as needed to provide the consumer more autonomy over their health data and information [75-77]. Access and control over personal health data are concepts similar to the “environmental factors” described by Tang and colleagues in their influential foundational PHR article [50]. Interoperability, lack of resources at the provider level, and PHR design and policy issues are factors outside the control of the individual participant and currently under the control of the provider or health care system. First, the decision makers in health systems or delivery systems should create policies to facilitate the correction of incorrect or missing data in the PHR. These policies would empower patients to work with their providers to assure the patient record is correct and current. Some health care organizations have had positive experiences with open access for consumers to the entire EHR/PHR including the provider notes [78]. Again, this is a policy issue that may promote productive interactions, engagement, and mutual trust.

Health system leaders can improve access issues for consumers by promoting policies that will encourage providers to release results sooner. Meaningful Use policy is currently encouraging more implementation of EHR/PHRs. Health care leaders are working on integration and interoperability of these records across health care systems and among individual providers [79].

### Self-Management Support Enhancements

A review of the literature helped the authors to identify the core ideas of patient engagement and health self-management, empowered individuals, and the tools and knowledge to impact their own health. Research on PHRs shows improved patient engagement essential for self-management support [80]. The PHR encouraged engagement by facilitating preparation for appointments, tracking of laboratory results and diagnostic studies, encouraging involvement in preventive care and screening, and encouraging consumers to suggest a course of treatment with their providers [81,82].

The informed, “activated” patient is a key component of the CCM. Patient activation is the level of skills, knowledge, and confidence a person has in managing one’s own chronic illness [83]. The highly activated patient therefore is engaged, informed, and confident in their ability to self-manage their own condition [84]. Hibbard, Stockard, Mahoney, Tusler [85] developed a Patient Activation Measure (PAM) to determine the levels of patient activation. The use of a PHR can increase patient activation. An RCT of patients assigned a PHR as an intervention resulted in higher PAM scores compared to the control group, especially with those who started with lower scores [86]. Additional eHealth components such as telehealth and mHealth applications may also be useful in self-management support and to promote patient engagement [33,87]. The findings suggest that the use of a PHR can promote an informed, activated patient and augment the CCM in the areas of self-management support and productive interactions.

### Clinical Decision Support Enhancements

Originally, the CCM identified clinical decision support (CDS) as a method to assure providers had access to the most current evidence-based clinical guidelines, protocols, and standard of care [67]. A study of the literature suggests the eCCM component of CDS should incorporate patient/consumer specific needs as follows: (1) visual access to data, (2) access to protocols, (3) care standards and evidence for self-management, (4) info buttons that access clinical guidelines, and (5) reminders for both the patients and providers. An RCT by Holbrook et al [88] found that people with type 2 diabetes had better outcomes when their intervention included a Web-based clinical decision support system shared by the patient and provider. Fox [22,23] found that chronically ill adults frequently go online to health sites to help make decisions about self-management of their condition. The Institute of Medicine [13,89,90] in three separate reports recommends incorporating eHealth tools for the promotion of CDS for both patients and providers to improve safety and self-management support.

### Clinical Information Systems Enhancements

Originally, the clinical information systems (CIS) element of the CCM primarily focused on registries, databases, and systems in place to support the access to protocols and current standards of care. With rapid expansion of the eHealth components of the EHR/PHR, partially due to Meaningful Use implementations, the opportunity exists to engage with and evaluate these tools as part of the CCM. In 2001, 18% of provider offices had implemented EHRs; that number is up to 78% as of 2013 and about half of those implementations are meeting the Meaningful Use criteria [91]. Tethered patient portals/PHRs are part of the Meaningful Use stage II requirements and are dependent on EHR implementations to be a useful part of the enhanced CCM [50]. Other eHealth components such as telehealth and mobile devices are also on the rise. The inclusion of such tools in the CCM as part of the CIS element is a logical next step and one that has been proven to improve health outcomes [14]. Like the growth of PHRs, mHealth apps and mobile phones are expected to grow at a rate of 47.6% over the next six years [92].

### Addition of eHealth Education to the CCM

Based on findings in the literature, an additional suggested major enhancement to the CCM is the addition of the support element “eHealth Education” (Figure 2). With health systems offering eHealth tools and consumers seeking eHealth solutions, providing the chronically ill adult with eHealth skills is needed [13]. Health literacy is essential for eHealth. Low health literacy is a long-standing problem in the current health care system [13]; 90 million Americans have poor health literacy—trouble understanding and managing their own health [93]. In a systematic review of eHealth interventions, Jacobs et al (2014) found that it is feasible to use eHealth tools to improve overall health literacy. Older adults make up the vast majority of those with chronic illness. And, while older adults are increasing their use of the Internet, social networking, and mobile phones, there is a gap in the literature in the evaluation of eHealth literacy for the older adult [94]. Choi and DiNitto studied older adults who are home-bound or poor and found opportunities for providing equipment and training that may improve eHealth literacy [95].

One component of health literacy that is problematic for many is health numeracy—people’s ability to understand numbers and mathematical principles in the management of their health care [96]. Findings by Lipkus, Samsa, and Rimer [97] also noted that highly educated individuals had numeracy problems. To compound this issue, eHealth tools give patients even more access to data, information, and knowledge that may be confusing. eHealth is changing so rapidly that researchers are recommending we re-assess how we should measure eHealth literacy including its numeracy component [98].

Training for both consumers and providers in how to construct and send Web-based and text messages that promote productive interactions may prove useful. Training on the selection and use of health-related websites may also prove useful. Adults are already using the Web to look for health information on the Internet and were not informed on how to identify who is providing the information and how to assess the quality of the information [99,100]. With the rapid expansion of mHealth apps for mobile phones, training on how to choose apps that can promote health outcomes may be needed for both patients and providers [101,102].

Training in eHealth is proven to increase confidence and self-efficacy in using the tools but training lags behind in the roll-out of new technologies to the general public [15,103]. In fact in telehealth applications, the lack of training was reported frequently as a barrier to use [15]. In a mixed-methods study, low-income patients with human immunodeficiency virus (HIV) who were trained to use the PHR had better self-efficacy, patient activation, and disease knowledge at follow-up [104]. To promote productive interactions between the informed, activated patient and the prepared, proactive practice team, the authors of this paper would also recommend provider team training in the use of eHealth tools. In a study involving randomly assigned group practices, the providers trained to use the eHealth tools had measurable changes in the effectiveness of their information management skills [105]. Training for both consumers and providers may improve the efficacy of the use of eHealth tools and should be considered in future research.

### Communication and the Addition of the Complete Feedback Loop (CFL)

Findings in the literature show that interventions that include the complete feedback loop are required for technology to promote improved health status in the chronically ill [14]. A key factor in the CCM is productive interactions between patients and providers. The activated patient is best suited to participate in the cooperative effort. In a qualitative study among chronically ill patients with a variety of levels of patient activation, it was found that being in control and working in a cooperative partnership with the provider was consistent with those patients who had the highest PAM scores [84]. This finding is similar with Yellowlees’ [106] definition of “mutual participation” where patients work in an equal, trusting, and cooperative manner using the Internet to facilitate communication (p. 117). The secure patient-provider email messaging portal in a PHR is an area where this mutual participation and cooperation will occur. A cross-sectional study found that patients who were working cooperatively with their providers and setting very specific and concrete goals and focusing on self-monitoring had much higher PAM scores [107]. The PHR, mobile devices, and text messaging are eHealth tools that can give consumers control over the timing and content of their messages.

Provider response times to messaging are very important to patients [108,109] and can negatively affect CFL communication cycle. Patient satisfaction with provider response times to patient messages and requests have been studied. One study noted that patient satisfaction using a patient portal email system is positively affected by shorter message response times [108]. Reti et al found that response times varied across health care organizations and that usual patient portal email messaging response times varied from 24 to 72 hours [110]. With the importance of the CFL for self-management support and productive interactions, we recommend enhancing the CCM with the formal insertion of the CFL into the model. Perhaps the visual representation of the CFL in the model surrounding the productive interactions will remind researchers and developers of eHealth tools they need to include this element into their interventions and research (see Figure 2).

## Discussion

### Principal Findings

The purpose of this paper was to use Theory Derivation process to review the chronic care and eHealth literature and to articulate how the CCM could be expanded to include eHealth tools. The research is clear in showing that eHealth technologies related to a variety chronic conditions can be used to enhance self-management and revise the CCM [87,111]. The evidence also suggests that eHealth tools can support productive patient-provider interactions and improve health outcomes [112,113].

This review and model development highlights several gaps in the literature. First, clear definitions for eHealth, telehealth, and PHRs are needed to move forward in formulating appropriate research questions. Second, a gap in the literature exists in the efficacy of using online health communities for self-management support. While social support itself is shown to improve engagement and health outcomes with adults who have chronic illness [114,115], little is known about whether social support offered in online health communities has the same effect. A review of the literature and research in this area are needed. Third, research findings where elements of the CCM were used in conjunction with the CFL need to be identified and evaluated. Jimison et al (2008) identified that eHealth interventions that included the CFL improved outcomes [14]. Greenwood, Young, and Quinn found that telehealth interventions for people with diabetes is the kind of eHealth intervention that can foster a CFL [33]. The CFL as it relates to eHealth interventions is an important component to assure the success of eHealth interventions and will require future research [14]. Last, health education and technology experts are needed to develop a curriculum to train patients/consumers to use the eHealth tools that have been shown to improve health outcomes for the chronically ill person. Additionally, health care providers will need training on how to implement eHealth interventions and how to educate their chronically ill patients to use these tools.

### Limitations

Limitations of this Theory Derivation process started with the fact that an exhaustive literature review was not completed for each of the eHealth components or the new elements added to the eCCM. Additionally, new and important literature is being added daily and with the scope of this project being so large, focused attention to the new knowledge was difficult to track. The opportunity exists for researchers to now concentrate on systematic reviews of the literature and conduct research specifically focusing on the individual components of the new eCCM model.

### Conclusion

In conclusion, there is strong evidence demonstrating that eHealth tools can strengthen and enhance the already successful CCM. Research to explicitly test the new eCCM and its components is the logical next step.

## Abbreviations

A1C: hemoglobin A1C
ACA: Affordable Care Act
AHRQ: Agency for Healthcare Research and Quality
CCM: Chronic Care Model
CDS: clinical decision support
CFL: complete feedback loop
CIS: clinical information system
DIKW: Data, Information, Knowledge and Wisdom Model
DOC: diabetes online community
eCCM: eHealth Enhanced Chronic Care Model
EHR: electronic health record
HITECH: Health Information Technology for Economic and Clinical Health Act
HIV: human immunodeficiency virus
IOM: Institute of Medicine
PAM: patient activation measure
PHR: electronic patient health record/patient portal
RCT: randomized controlled trial
